# Portable telepathology: methods and tools

**DOI:** 10.1186/1746-1596-3-S1-S19

**Published:** 2008-07-15

**Authors:** Luis Alfaro, Ma José Roca

**Affiliations:** 1 Department of Pathology. Hospital Rey Don Jaime. Castellón Spain; 2 Department of Pathology. Hospital Lluis Alcanyis. Xativa. Valencia, Spain

## Abstract

Telepathology is becoming easier to implement in most pathology departments. In fact e-mail image transmit can be done from almost any pathologist as a simplistic telepathology system. We tried to develop a way to improve capabilities of communication among pathologists with the idea that the system should be affordable for everybody. We took the premise that any pathology department would have microscopes and computers with Internet connection, and selected a few elements to convert them into a telepathology station. Needs were reduced to a camera to collect images, a universal microscope adapter for the camera, a device to connect the camera to the computer, and a software for the remote image transmit. We found out a microscope adapter (MaxView Plus) that allowed us connect almost any domestic digital camera to any microscope. The video out signal from the camera was sent to the computer through an Aver Media USB connector. At last, we selected a group of portable applications that were assembled into a USB memory device.

Portable applications are computer programs that can be carried generally on USB flash drives, but also in any other portable device, and used on any (Windows) computer without installation. Besides when unplugging the device, none of personal data is left behind. We selected open-source applications, and based the pathology image transmission to VLC Media Player due to its functionality as streaming server, portability and ease of use and configuration. Audio transmission was usually done through normal phone lines. We also employed alternative videoconferencing software, SightSpeed for bi-directional image transmission from microscopes, and conventional cameras allowing visual communication and also image transmit from gross pathology specimens.

All these elements allowed us to install and use a telepathology system in a few minutes, fully prepared for real time image broadcast.

## Introduction

Telepathology is closely tied to advances in communications technology. There is already a long run from its beginning, and huge changes have occurred since the first developments. Cost and complexity of equipment limited the number of pathologists interested and institutions implementing the equipment. The Internet era has been the revolution that simplified communications and overcame the problem of installing specialized lines to communication equipment. Prices are now much more affordable than only a few years ago [1,2]. However, telepathology still remains confined to a limited number of pathologists. New tools have come with virtual slides and sophisticated scanners which are not available for everybody, but telepathology can be reliable with very simplistic elements [3,4].

We tried to develop a way to improve capabilities of communication among pathologists with the idea that the system shouldn't be focussed on telepathologists with especial interests or previous experience in digital pathology, but on any pathologist regardless of working conditions. We took the premise that any pathology department would have microscopes and computers with Internet connection and we selected a few elements to convert them into a telepathology station. They should achieve the goals of being light and easily portable, simple to install in a short time, effortless to use, and universal in the potential to adapt to any place. We didn't intend to develop image transmit systems for wireless connections, or mobile computers or devices, but to connect any pathology department in the easiest way.

Needs were reduced to a camera to collect images, a universal microscope adapter for the camera, a device to connect the camera to the computer, and software for the remote image transfer.

## Materials and methods

We decided to employ domestic digital cameras based on their low price, good resolution, and wide circulation. They also needed to have a video output for connection to TV and computers, lens thread to fix on the microscope, and a minimum 3× zoom to solve focusing length in different microscopes. We selected three cameras:

1. Nikon Coolpix P5100 with a 1/1.8", 12 Megapixel CCD sensor and 35–123 mm (3.5×) zoom lens.

2. Olympus SP-350 with a 1/1.8", 8 Megapixel CCD sensor and 38–114 mm (3×) zoom lens.

3. Pentax Optio S7 with a 1/2.5", 7 Megapixel CCD sensor and 37.5–112.5 mm (2.9×) zoom lens.

The Nikon camera used a Nikon UR-E20 Converter Adapter to get a 37 mm mount. The Olympus camera used an Olympus CLA9 Lens Adapter tube to get a 37 mm mount. The Pentax S7, an ultracompact camera with a weight of only 120 g, lacked lens thread and needed a special L adapter (Pentax PF-DS1) to get a 43 mm mount. The camera connections to the microscope was achieved using a universal adaptor MaxView™ Plus System [5]. This kit includes threads to fit optical devices with any the following attachments; C-Mount, C/S-Mount, T-Mount, 23 mm Eyepiece Port, 30 mm Eyepiece Port, and 1.25" Eyepiece Ports, therefore being suitable for almost any microscope even without a triocular head. Intermediate step up/down rings (43–52 and 37–52) were used to connect cameras to MaxView, and are easily available [6,7].

As most computers do not have video capture cards installed, we chose an external device for this purpose which was easy to install and to move with all the telepathology elements. Video signal from the camera was sent to the computer through an AverMedia Hybrid USB stick, not much bigger that conventional USB memories.

Finally, software for image and real time video telepathology sequences broadcast was selected from the so-called portable applications [8]. These programs can be used in any Windows computer, do not need any installation, and do not leave any data after switching off. USB flash drives are optimal to move them from computer to computer. VLC Media Player portable was the main application employed [9]. Besides being a free multiformat video and audio player, it can also be used as a server to stream in unicast (one to one machine) or multicast (from one to a dynamic group of machines that the clients can join or leave) [10]. Program configuration let selecting the video source, in our case microscope attached camera connected to Aver Media capture device, and choose streaming method, usually with UDP protocol for unicast when knowing client's IP address, or with HTTP protocol in which the program listens to any network interfaces on port 8080.

Effectiveness of the image transmit will finally depend on the bandwidth available for Internet connection. We were employing most of the time ADSL (Asymmetric Digital Subscriber Lines) between 1 and 3 Mbps.

We also employed alternative videoconferencing software, SightSpeed, for bi-directional image transmition from microscopes, and conventional cameras allowing audio and visual communication, and also image broadcast from gross pathology specimens.

## Results

The server equipment was prepared to work in several pathology departments from different hospitals. A few minutes were enough to arrange all the components, and to provide video sequences in real time with sufficient quality for normal remote diagnosis. Receptors obtain images without any special equipment, in any Internet connected computer. Most of the time there was no need to implement audio connections because conventional phone calls were available for communication between server and receptors, and in this way all the network bandwidth can be dedicate to achieving maximum image quality, and receptors do not need to install special software.

The biggest trouble we found putting the equipment into practice was security restrictions from the net administrators that most hospitals and institution have. It's very common that computers at pathology departments have limitations to connecting freely outside the institution. Internal IP addresses situated behind firewalls and proxies cannot be reached from outside, and ports are frequently blocked. The only way to solve these situations is to get in touch with net administrators and obtain permit to translate external IP addresses into our computer, and open necessary ports.

## Discussion and conclusion

Portable telepathology can be a very useful, cost effective, and easy to implement method to increase telepathology equipments. Simple and effortless solutions can reach the smallest and most poorly equipped pathology departments. Limitations for telepathology systems were the high cost of equipment and lack of universality. The Internet has dramatically changed telepathology possibilities in means of universality. Now price is not necessary a limitation. The goal for the future can be to change the idea of telepathology as a highly technicalized subspecialty, and reduce it to a new and broad and fully open way of communication among pathologists
